# Automatic Calibration of the Adaptive 3D Scanner-Based Robot Welding System

**DOI:** 10.3389/frobt.2022.876717

**Published:** 2022-05-24

**Authors:** Peter Arko, Matija Jezeršek

**Affiliations:** ^1^ Yaskawa Slovenija, Ribnica, Slovenia; ^2^ Laboratory for Laser Techniques, Faculty of Mechanical Engineering, University of Ljubljana, Ljubljana, Slovenia

**Keywords:** 3D scanner calibration, robot welding system, automatic calibration, laser triangulation profilometer, adaptive robot control

## Abstract

An advanced automatic calibration procedure and its versatile usage in the context of the adaptive robot welding technology are presented. The 3D scanner-based robot welding system calibration is composed of the measurement of the reference plate and numerical optimization of the hand-eye and intrinsic parameters by minimizing the deviation between the measured and reference plate. The measurements of the reference plate are acquired from various robot poses (typically 15). The shape features of the reference plate are then detected, and finally, the calculation of hand-eye and intrinsic parameters is performed using Powell’s optimization algorithm, where the merit function presents an average deviation between the measured and reference geometry. Validation experiments show appropriate system accuracy which is better than 0.06 mm perpendicular to the scanning direction. This calibration procedure’s important features are complete automation and fast execution times (approximately 90 s). This enables its implementation into a regular daily robot self-maintenance and monitoring plan. The universal use of such a robot welding system is demonstrated in multi-layer heavy-duty welding of thick pipes on cast machined hollow parts and in precise laser welding of thin sheet metal parts.

## Introduction

Robotic welding is an important production technology that significantly increases the precision and efficiency of welding in numerous fields of industry (Shimon et al., 2020). Workpiece complexity and small series of products are driving the development of this technology to shorten the programming time of the welding paths and enable in-line robot movement adaptation due to various influences, such as deviations between a CAD model and a real part, or thermal distortions of the workpiece (Jezeršek et al., 2017; Kos et al., 2019). A promising solution to these issues is the integration of a laser profilometer onto the robot arm, which measures the shape of the workpiece in the vicinity of the weld path and corrects the robot movement accordingly. A simple measurement principle, usually based on the laser triangulation principle (Dorsch et al., 1994), offers a compact and robust design, enabling their wide application. In addition to adaptive robotic welding (Hatwig et al., 2010; Jezeršek et al., 2017; Kos et al., 2019; Lee et al., 2020), other types of adaptive machining can be performed efficiently, such as milling (Pérez et al., 2016) and deburring (Kosler et al., 2016).

The robot arm moves its welding torch together with the laser profilometer in all six degrees of freedom and to measure the workpiece shape; a transformation of the acquired profiles into a global coordinate system must be performed by knowing the accurate pose of the profilometer relative to the robot arm. This involves a so-called hand-eye calibration (Tabb and Ahmad Yousef, 2017; Liska et al., 2018). Furthermore, the profilometer itself must also be calibrated to determine its intrinsic parameters, such as focal length, optical distortions, and laser pose relative to the camera. The calibration procedures are usually composed of two separate steps: i) intrinsic and ii) hand-eye calibration (Heikkilä et al., 2014; Idrobo-Pizo et al., 2019). The relationship between the robot arm and the sensor coordinate system is reported as the hand-eye rotation matrix (X), which is defined by the AX = XB equation, where A represents the transformation between each robot arm position and the B transformation between each camera position. The method (Deniz and Cakir, 2017b; 2017a) represents the mentioned equation in quaternion representation, solving an absolute orientation problem. Hand-eye calibration often mimics the well-known method of Tool Center Point (TCP) determination (Handbook of Industrial Robotics, second Edition | Wiley) by touching a fixed point either mechanically with an end-effector from different orientations (Cakir and Deniz, 2019), where a novel method of automatically calculating TCP using plane equations is introduced. The method is based on detecting the contact between a robot tool and a flat, electrically conductive plate. Alternatively, an optical approach using external sensors (Gordić and Ongaro, 2016; Luo and Wang, 2018), or a profilometer itself, can be used (Li et al., 2011; Liska et al., 2018). However, these methods require special algorithms for recognizing the reference shape and simultaneous movement of the robot around this object. Most importantly, these methods do not calibrate the intrinsic parameters of the laser profilometer. Thus, in the case of a profilometer repair, an additional calibration must be carried out.

The intrinsic calibration is typically based on measuring a reference 2D plate with a circular grid (Wu et al., 2016), holes (Idrobo-Pizo et al., 2019), or checkerboard (Tsai, 1987), which is acquired in various poses. However, to acquire precise images of these plates, additional illumination must be used together with additional image processing algorithms for 2D pattern detection. Therefore, the most advanced calibration approaches include the determination of all transformations in a single step (Jezeršek, 2009; Santolaria et al., 2009; Novak et al., 2014; Wang et al., 2020; Pavlovčič et al., 2021). The method in Wang et al. (2020) uses the same set of calibration images for the profilometer and hand-eye calibration, but the calculation itself is divided into several distinctive steps. In Santolaria et al. (2009), the profilometer and hand-eye calibration are calculated in a single step, after mounting the profilometer to an articulated arm coordinate measuring machine and referencing the local reference frame of the calibration object. The methods in Jezeršek (2009), Novak et al. (2014), and Pavlovčič et al. (2021) introduce the calibration of the laser-scanning apparatus based on measuring a single reference 3D geometry with a series of perpendicular grooves. All the transformation parameters are then numerically optimized by minimizing the average deviation between the measured and the reference geometry. This allows the use of the same scanning procedure and detection algorithms as in normal operation. Its drawback is the requirement of relatively precise knowledge of initial guess values of transformation parameters; otherwise, the numerical convergence is slow or can reach a wrong local minimum.

This study presents further development of the recent method for the simultaneous calibration of hand-eye and intrinsic parameters (Pavlovčič et al., 2021). The new calibration method uses a reference geometry, which enables the determination of deviations between the measured and the reference geometry in all three spatial dimensions. This significantly improves the robustness of the transformation parameter calculation, which is based on numerical optimization of the abovementioned deviation and therefore results in faster and more precise calibration. Additionally, the developed method avoids the use of approximated techniques to access the hand-eye and reference object robot relations, after assembling the sensor on the robot arm.

The following content is divided into five sections. The experimental system is described in the first section, automatic calibration procedure in the second section, validation experiments on lap seam geometry are shown in the third section, where the influence of the scanning angle, on repeatability and accuracy characterization of detected edge is presented. The fourth section demonstrates the versatile uses of such a robot welding system in multi-layer heavy-duty welding and precision laser welding.

## System Description

**FIGURE 1:**
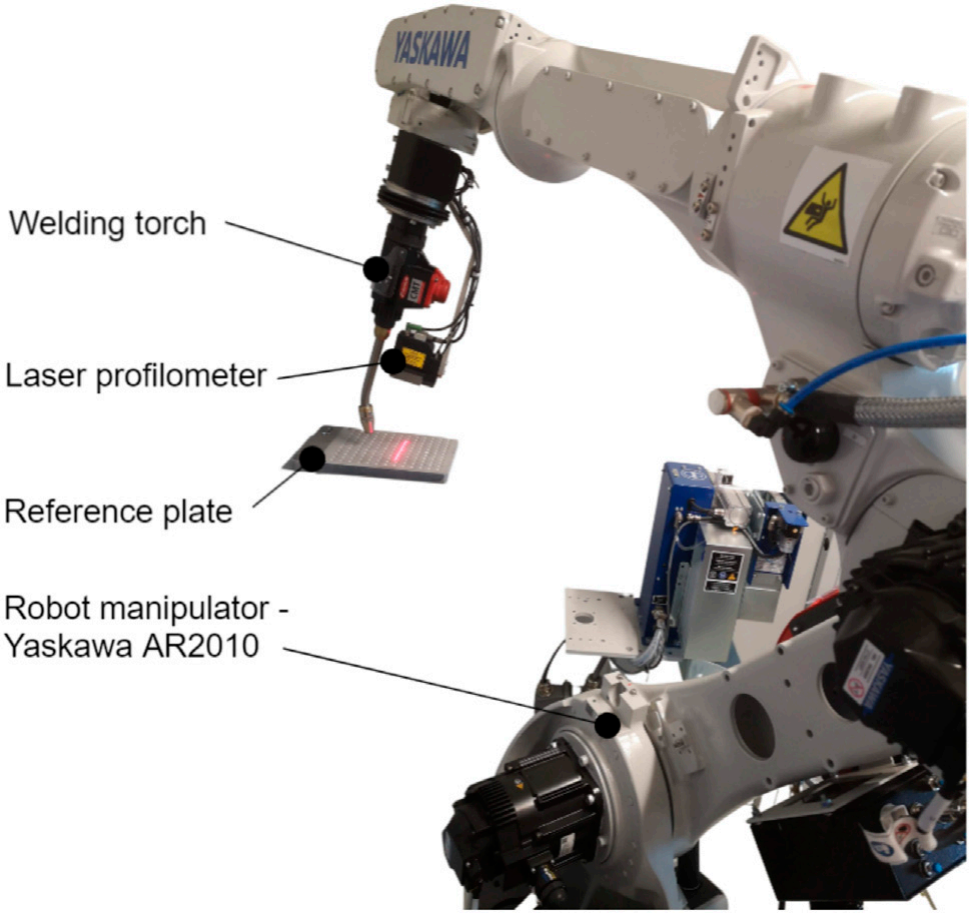
Robotic welding system with a laser profilometer during the measurement of the reference plate that is used for system calibration.

The presented adaptive 3D scanner-based robot welding system consists of three main components (see Figure 1): a laser profilometer (Yaskawa MOTOSense, Ribnica, Slovenia), a six-axis industrial welding robot (Yaskawa AR2010 and Yaskawa MC 2000, Fukuoka, Japan) with a corresponding controller (Yaskawa YRC1000 and Yaskawa DX200, Fukuoka, Japan), and a reference plate used for calibration.

The measuring range of the laser profilometer is from 58 to 435 mm, resolving acquired profile widths from 25 mm to 136 mm. The precision of the profilometer is 0.02 mm at the closest working distance and 0.05 mm at the most frequently used distance of 150 mm. A USB 3.0 camera (manufacturer XIMEA, Germany, model xiQ MQ013MG-ON, resolution 1,280 × 1,024 pixels, 210 frames per second at full resolution) inside the laser profilometer captures images of the surface illuminated by the laser line projector (manufacturer Laser Components, Germany, model Flexpoint MVpico, wavelength 660 nm), which is also a part of the profilometer.

Simultaneously, with each image, a robot pose is acquired from the robot controller, and both are fed into an industrial computer (Intel Core i7-7700HQ, 2.80 GHz processor, and 16 GB of RAM). A custom-developed LabView-based software (manufacturer: National Instruments, United States) communicates with the camera and the robot controller and processes the acquired data to detect the profiles with a subpixel resolution from each image and further transform them into a 3D space.

### Mathematical Formulation of 3D Transformation

**FIGURE 2:**
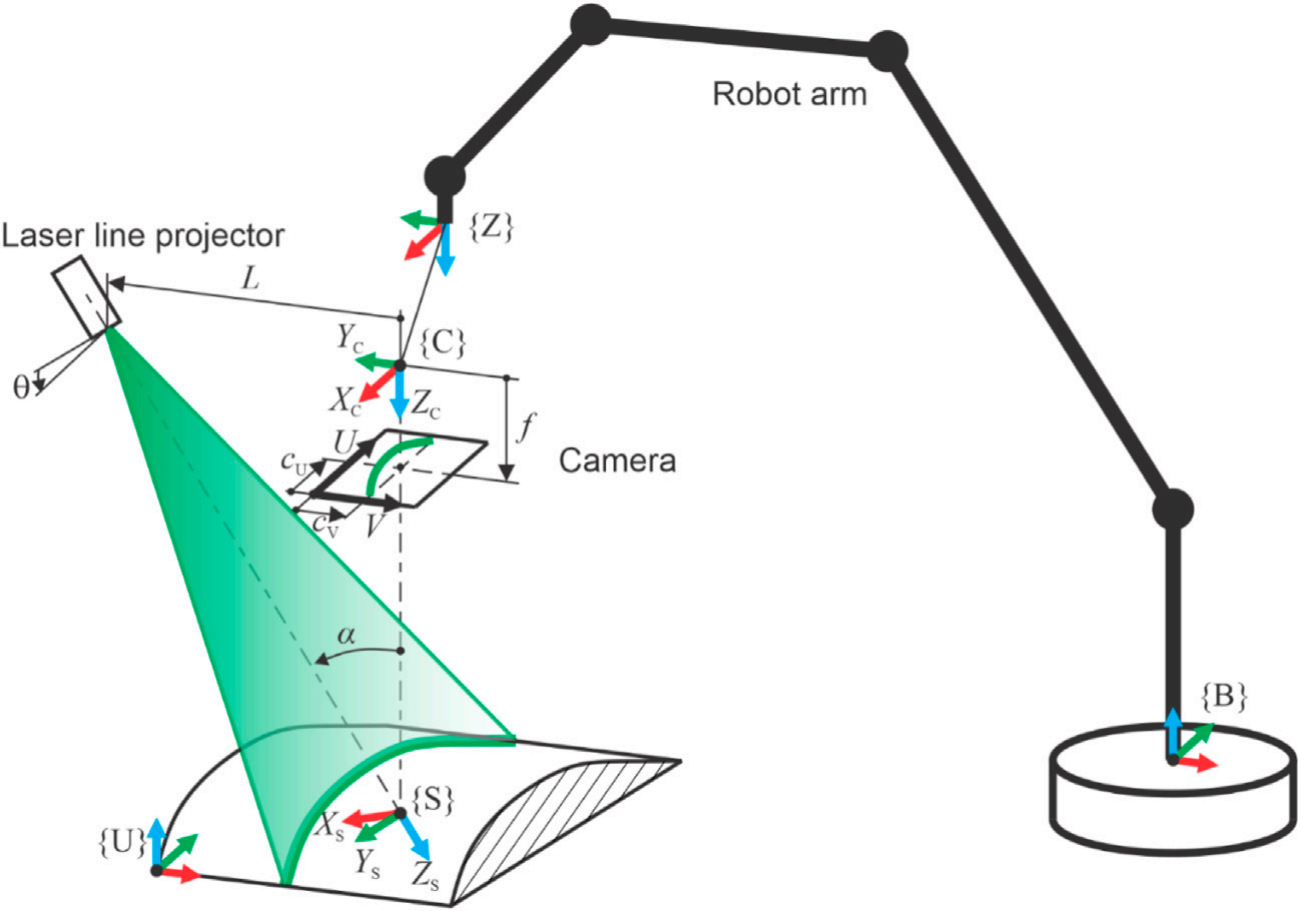
Schematic representation of the 3D measurement principle based on laser triangulation, where the required scanning movement is performed by the robot arm while holding the laser profilometer. Relevant coordinate systems (c.s.) are as follows: robot base c.s. {B}, robot zero c.s. {Z}, sensor c.s. {S}, camera c.s. {C}, and user c.s. {U}.

The three-dimensional measuring principle is based on laser triangulation and scanning by the robot arm, as is illustrated in Figure 2. The camera captures an intersection contour between a measured surface and a laser light plane, which is generated by a laser line projector. The contour is determined by the array of points with coordinates *u* and *v* in the image coordinate system.

To transform these points into a final user coordinate system {U}, a lens distortion is corrected in the first step (Brown, 1971):

$$u_{\text{UD}}=u+\left(u-c_{\text{U}}\right)\left(k_{1}r^{2}+k_{2}r^{4}\right)+p_{1}\left(r^{2}+2{\left(u-c_{\text{U}}\right)}^{2}\right)+2p_{2}\left(u-c_{\text{U}}\right)\left(v-c_{\text{V}}\right),$$ (1)

$$v_{\text{UD}}=v+\left(v-c_{\text{V}}\right)\left(k_{1}r^{2}+k_{2}r^{4}\right)+2p_{1}\left(u-c_{\text{U}}\right)\left(v-c_{\text{V}}\right)+p_{2}\left(r^{2}+2{\left(v-c_{\text{V}}\right)}^{2}\right),$$ (2)

where *c*_U_ and *c*_V_ are coordinates of the principal point on the camera’s sensor, *k*_1_ and *k*_2_ are radial distortion coefficients, and *p*_1_ and *p*_2_ are tangential distortion coefficients. The normalized coordinates *u*_N_ and *v*_N_ are then calculated (Jezeršek, 2009):

$$u_{\text{N}}=\frac{\left(c_{\text{U}}-u_{\text{UD}}\right)\cdot d_{\text{U}}}{f},$$ (3)

$$v_{\text{N}}=\frac{\left(c_{\text{V}}-v_{\text{UD}}\right)\cdot d_{\text{V}}}{f},$$ (4)

where *f* is the focal length of the camera lens, and *d*_U_ and *d*_V_ are the sensor pixel dimensions. Transformation into the camera’s coordinate system {C} is calculated by the following set of equations:

$$Z_{\text{C}}=\frac{L}{v_{\text{N}}+\tan\left(\alpha \right)},$$ (5)

$$X_{\text{C}}=Z_{\text{C}}\cdot v_{\text{N}},$$ (6)

$$Y_{\text{C}}=-Z_{\text{C}}\cdot u_{\text{N}},$$ (7)

where *L* is the distance between the projector and the camera in the *Y*_C_ direction, and *α* is the triangulation angle between the projector’s and the camera’s optical axes. The transformation of points from {C} to {S} is then calculated:

$$P_{S}=RT_{C-S}\cdot P_{C},$$ (8)

where $P_{\text{C}}={\left[\begin{array}{cccc} X_{\text{C}} & Y_{\text{C}} & Z_{\text{C}} & 1 \end{array}\right]}^{T}$ and $P_{\text{S}}={\left[\begin{array}{cccc} X_{\text{S}} & Y_{\text{S}} & Z_{\text{S}} & 1 \end{array}\right]}^{T}$ are point vectors, and **RT**_C-S_ is a homogeneous transformation matrix from {C} to {S}:

$$RT_{\text{C}-\text{S}}=\left[\begin{array}{cccc} \cos\left(\theta \right)\cos\left(\alpha \right) & -\sin\left(\theta \right) & \cos\left(\theta \right)\sin\left(\alpha \right) & 0\\ \sin\left(\theta \right)\cos\left(\alpha \right) & \cos\left(\alpha \right) & \sin\left(\theta \right)\sin\left(\alpha \right) & 0\\ -\sin\left(\alpha \right) & 0 & \cos\left(\alpha \right) & \frac{Z_{\text{C}}\cdot L}{\tan\left(\alpha \right)}\\ 0 & 0 & 0 & 1 \end{array}\right],$$ (9)

where *θ* is an angle between the laser plane and the *Y*_S_ axis. The points are further transformed from {S} to {Z} and then into {B}:

$$P_{B}=RT_{Z-B}\cdot RT_{S-Z}\cdot P_{S},$$ (10)

where **RT**_Z-B_ and **RT**_S-Z_ are homogenous transformation matrices for transformations from {Z} to {B} and from {S} to {Z}, respectively. **RT**_Z-B_ represents the pose of the robot’s end-effector and is streamed from the robot controller as the current feedback position to the system computer simultaneously with each recorded image. Meanwhile, the **RT**_S-Z_ describes the pose of the laser profilometer relative to {Z}. Finally, the transformation into the user’s c.s. {U} is calculated:

$$P_{U}=RT_{B-U}\cdot P_{B},$$ (11)

where **RT**_B-U_ describes the pose of {U} relative to the base c.s. {B}.

The first nine equations represent transformations of contour points inside the profilometer, where 12 intrinsic transformation parameters must be known. Eq. 10 describes the so-called hand-eye transformation, which is determined by three translations X_S-Z_, Y_S-Z_, and Z_S-Z_ and three rotations RX_S-Z_, RY_S-Z_, and RZ_S-Z_. Similarly, in Eq. 11, another six parameters describe the transformation to user c.s. Altogether, 24 transformation parameters must be precisely calculated to achieve accurate 3D measurements.

## System Calibration

The system calibration determines the values of the transformation parameters while searching for the global minimum of the merit function, defined as the deviation between the measured and the reference (theoretical) shape of the reference plate. The calculation is performed using Powell`s optimization algorithm (Powell, 1964) to find the local minimum of the merit function. This function is upgraded from the one presented in Pavlovčič et al. (2021), where deviations were measured only in the vertical direction, in such a way that deviations are now decomposed in all three (X, Y, and Z) directions:

$$\mathrm{DEV}\left(C\right)=\sqrt{\frac{1}{N}\sum_{i=0}^{N-1}\left(\Delta X_{\text{Ref}}{\left[i\right]}^{2}+\Delta Y_{\text{Ref}}{\left[i\right]}^{2}+\Delta Z_{\text{Ref}}{\left[i\right]}^{2}\right)},$$ (12)

where *N* is the number of the reference points, **C** is the vector of the transformation parameters, Δ*X*_Ref_ [*i*], Δ*Y*_Ref_ [*i*], and Δ*Z*_Ref_ [*i*] are the deviations between the measured and reference *i*th point along each axis of the reference c.s, which is used during the calibration process instead of the user c.s.

**FIGURE 3:**
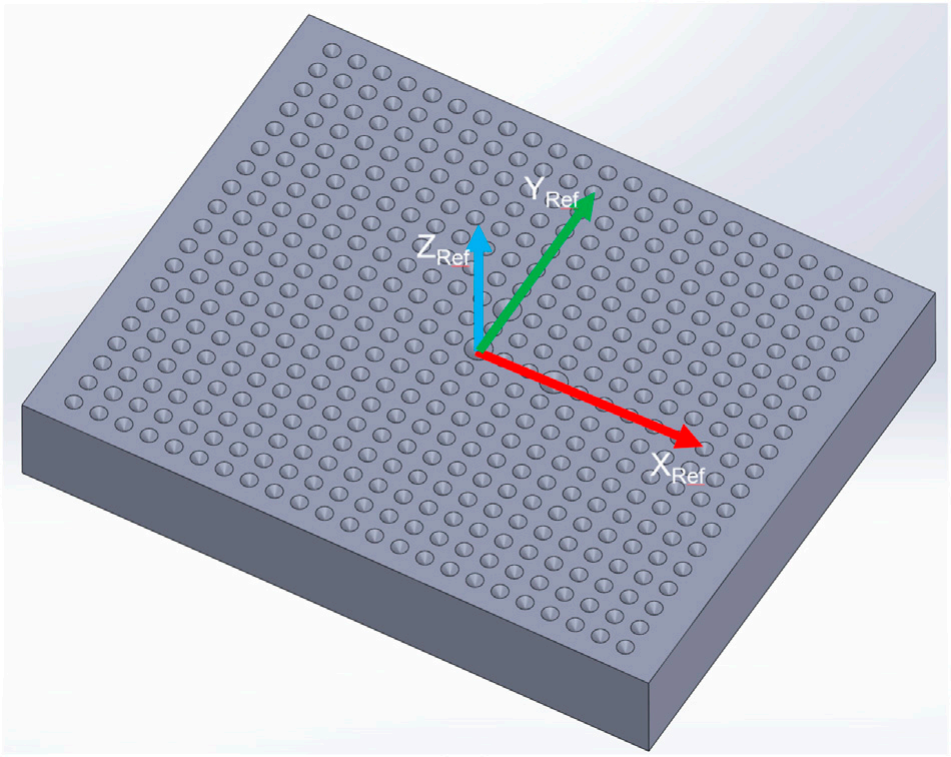
Geometry of the reference plate is composed of a matrix of conical holes where the central three holes determine the pose of the reference coordinate system (XRef, YRef, and ZRef).

The reference points are defined by conical holes which are drilled into a reference plate in a 2D 187 pattern (Figure 3). The center section includes three bigger holes, which indicate the origin of the reference coordinate system and its orientation. We are using two sizes of the reference plate, according to the required measuring range of the system. The smaller plate measures 100 × 80 × 15 mm in size, with cones at an angle of 120° and a diameter of 4 mm for the central ones and 2.5 mm for the rest. The number of holes is 437 in the raster of 4 mm. The bigger plate measures 210 × 185 × 20 mm in size, with cones at an angle of 90° and a diameter of 12 mm for the central ones and 8 mm for the rest. The number of holes is 143 in the raster of 15 mm. Both plates are made of the aluminum alloy EN AW-2007, which is milled with accuracy better than 0.005 mm. The surface was sandblasted after the milling process to assure diffuse light reflection.

**FIGURE 4:**
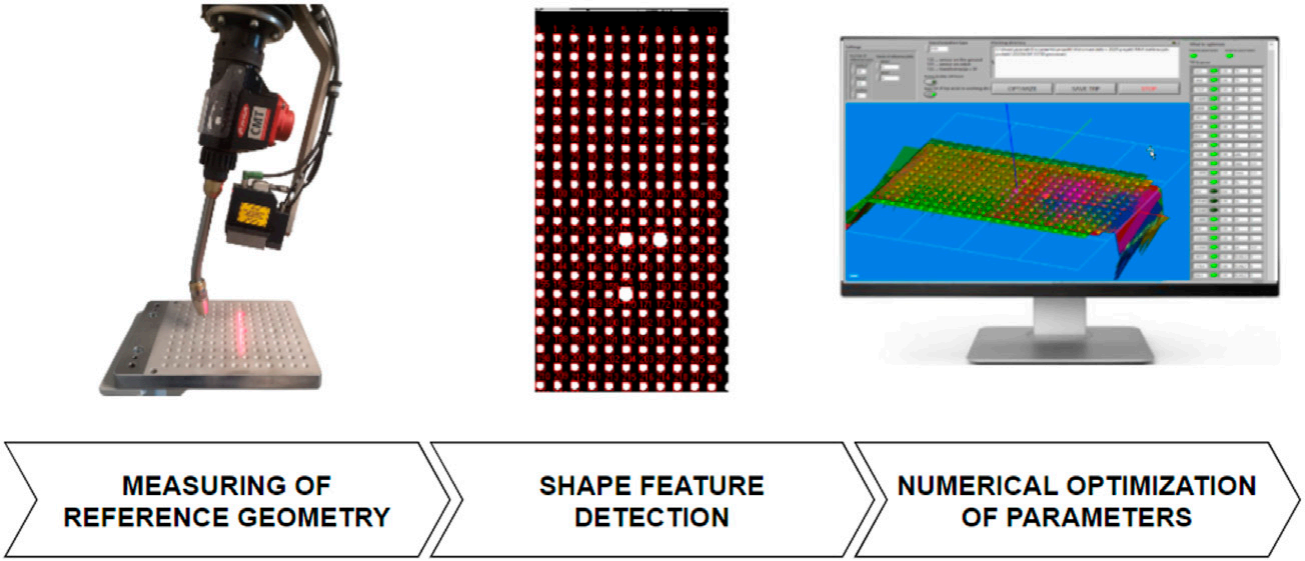
Calibration procedure consists of three basic steps: measuring the reference geometry, shape feature detection, and numerical optimization of 3D transformation parameters.

Three main steps of the calibration procedure are schematically presented in Figure 4. In the first step, the 3D measurements of the reference plate are acquired from 15 different poses, where three distances from the plate (−60 mm, focus distance, +60 mm) are combined with three rotations around the approximate X_Ref_ and Y_Ref_ axes defined prior to measuring. The scanning direction is approximately parallel to the X_Ref_ axis in all poses. By changing the robot orientation, we increased the sensitivity of the deviation between the reference and measured plate features to the hand-eye parameters. However, the angles were limited to ±10° to minimize the influence of the robot’s positioning inaccuracy.

The shape feature detection is performed on each scan in the second step, where the conical holes are automatically detected and indexed. The raw depth images are first segmented into flat areas and into the holes where their centers are used as reference points. Then, the bigger three holes are used for setting index values along the *X* and *Y* directions. To distinguish between both directions, the distance between bigger holes along the X direction is three increments, while along the Y direction it is only two increments. Finally, the reference points are stored in a database together with the robot poses. Thus, each of the 15 calibration measurements contains a table where rows consist of the coordinates (u, v, X_ref_, Y_ref_, and Z_ref_) of the detected points in the image coordinate system and in the reference coordinate system, respectively. The corresponding poses of the robot arm (X, Y, Z, Rx, Ry, and Rz) are appended to each row.

A calculation of the transformation parameters starts by reading the database of the reference points into the custom-developed calibration program of the MOTOSense software (Yaskawa, Slovenia). The initial values of the transformation parameters (intrinsic parameters) are estimated from the sensor’s geometry in product documentation and the specifications of the camera and lens. The merit function (Eq. 12) is calculated during numerical optimization by transforming the u and v coordinates of all reference points into a reference coordinate system. Then, the deviations between measured and reference values are calculated, and finally, the DEV is calculated.

Optimization of DEV is performed numerically by variation of all transformation parameters using Powell’s algorithm where the bracketing of the minimum DEV along the optimization direction (Press et al., 2007) is iteratively performed. Since Powell’s algorithm searches for a local minimum of DEV, the optimization is executed multiple times where the initial values of **C** for the next iteration are randomized. Typical values of randomization intervals are ±10% of the value of each transformation parameter. The newly optimized parameters are stored as the new optimum ( $C^{\text{opt}}$ ) if DEV is lower than any one of the previous iterations.

Convergence of the optimization is reached when the relative change of DEV is lower than the prescribed tolerance (typically ΔDEV < 0.02%), and its value is lower than the declared threshold (typically DEV_max_ = 0.1 mm). Transformation parameters are then stored in the system memory. Otherwise, if the convergence is not reached after the maximum number of iterations, a service warning is displayed to check the robot’s accuracy, clean the optics, set the image acquisition parameters, or refocus the optics of the camera and projector.

**FIGURE 5:**
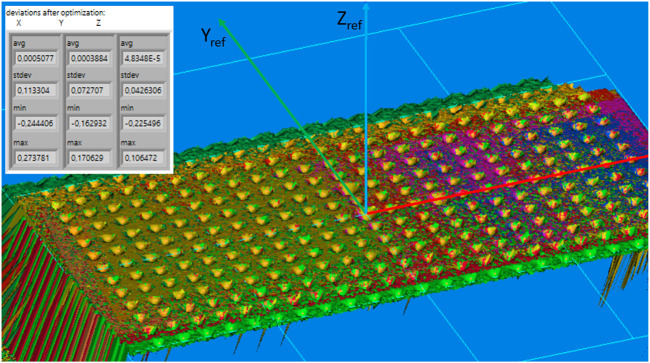
Typical result of the system calibration. The 3D image shows the reference plate where different colors represent scans measured from various poses (15 in total). The table shows statistics of the final deviations of the reference points in each direction.

Figure 5 shows the final result of the measured reference plate from various poses (marked with different colors, 15 in total) after the system calibration. In this example, the standard deviations of the reference points were 0.113, 0.072, and 0.043 mm along the X_ref_, Y_ref_, and Z_ref_ directions, respectively. A slightly higher deviation along the X_ref_ direction is due to the smaller repeatability of the robot’s movement in this direction and the jitter of the trigger signal between the robot and the camera. However, deviations in lateral directions (Y_ref_ and Z_ref_) are smaller than 0.08 mm, which demonstrates adequate accuracy for high-precision welding applications, where the accuracy perpendicular to the welding direction is the most important. The time needed for the entire calibration is approximately 90 s, whereas scanning takes approximately 70 s, and data processing takes approximately 20 s.

### Validation of Welding Edge Detection

After calibration of the transformation parameters, the system can be used for various welding applications, where the edge position must be located with high accuracy to position the welding torch in the right position. In this section, the accuracy and repeatability of the detected welding edge are validated for various poses of the laser profilometer relative to the part.

**FIGURE 6:**
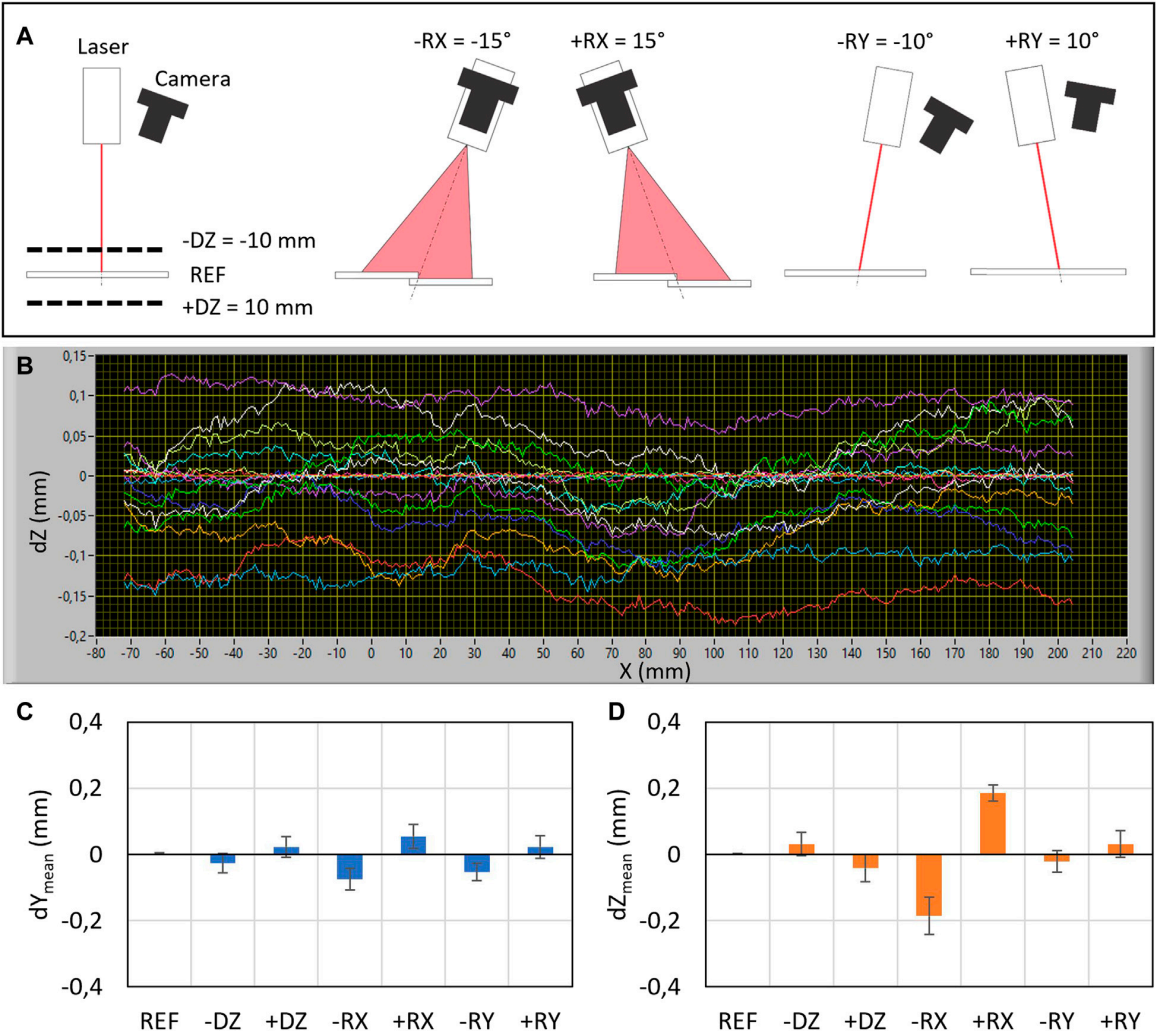
Results of welding edge detection in terms of repeatability and accuracy for various poses of the laser profilometer relative to the welding edge. **(A)** Schematic representation and names of examined poses (−DZ, REF, +DZ, −RX, +RX, −RY, and +RY). **(B)** Deviations of the measured edges in the vertical direction (dZ) for various poses are shown in different colors. Mean deviations for various poses along the Y direction **(C)** and Z direction **(D)**. Error bars represent repeatability for 10 consecutive measurements.

For that purpose, the lap weld geometry of two carbon steel plates (rolled plates) with dimensions 300 × 100 mm × 2 mm was used. Seven poses were tested (Figure 6A), where the reference one (REF) denotes the perpendicular orientation of the laser profilometer to the welding edge, while the measuring distance was 110 mm. At other poses, the measuring distance (−DZ, +DZ), inclination around the weld direction (−RX, +RX), and inclination around the Y-axis (−RY, +RY) were varied as is shown in Figure 6A. The weld edge along the length of 280 mm was measured 10 times for each pose (see Figure 6B).

Figures 6C,D show accuracies and repeatability (error bars) for each pose along the *Y* and *Z* directions. Repeatability was calculated as the average scatter of trajectories along their complete length for all measurements within each pose, while the accuracy was calculated as an average deviation of the detected trajectory relative to the reference pose. Results show that accuracy is better than 0.06 and 0.2 mm along the *Y* and *Z* directions, respectively. The highest impact on the accuracy has the inclination around the X-axis. Since the mean deviations are almost symmetrical for negative and positive inclinations, we assumed that the robot positioning uncertainty, which is in the range of 0.1 mm for typical industrial robots (Slamani et al., 2012), represents the most influencing factor.

## Application Examples

The applicability of the presented robot welding system is demonstrated in multilayer heavy-duty welding and precision laser welding.

### Multilayer Welding

**FIGURE 7:**
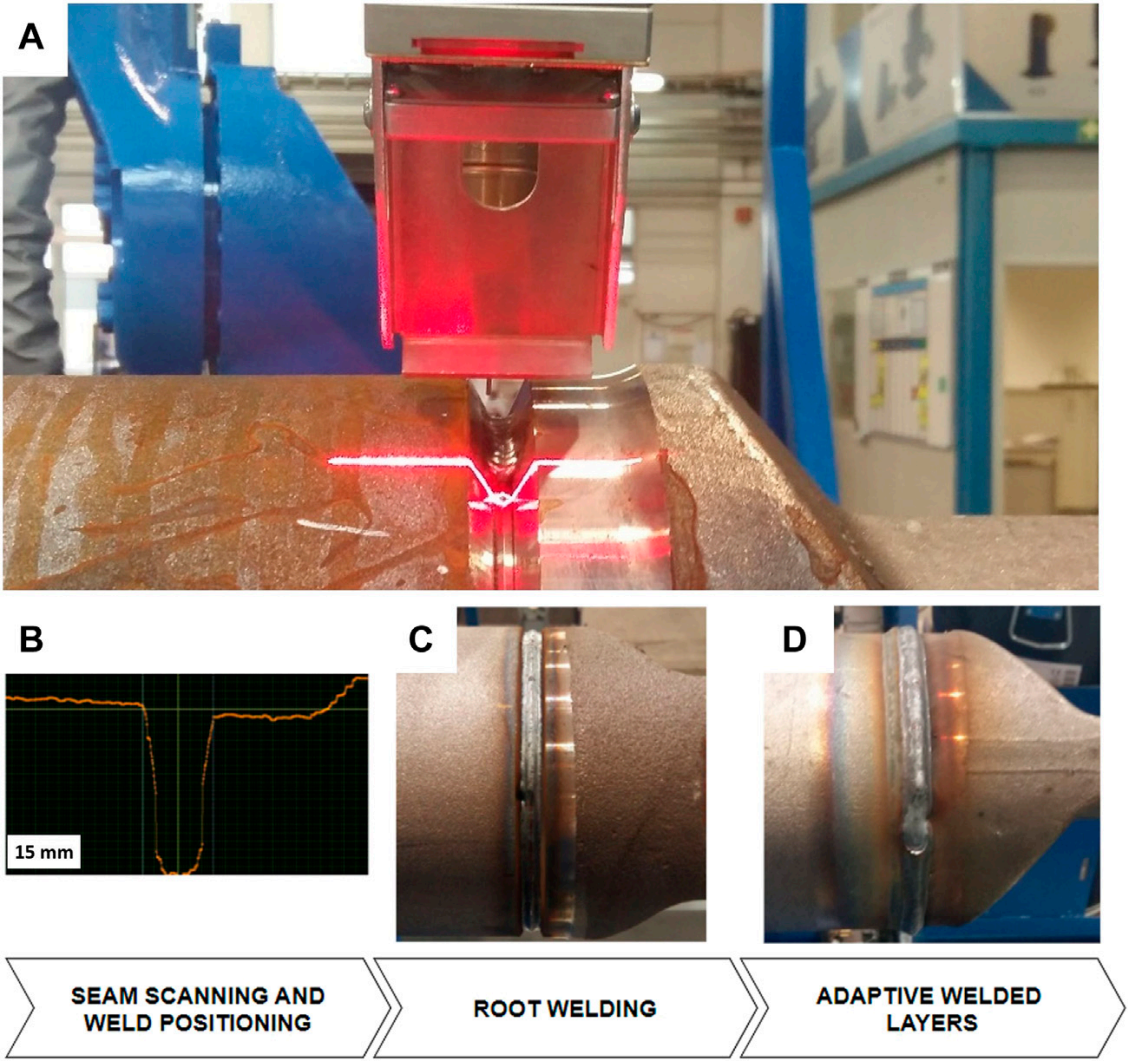
Example of adaptive multilayer welding where the welding area is first scanned in 3D **(A,B)**; then, a root weld is made **(C)**, and finally, multiple layers are adaptively added **(D)**.

Figure 7 shows an example of adaptive robotic arc welding (using Yaskawa robot MA1440 with a Fronius welding source and torch) of thick cast parts. The laser profilometer (Yaskawa MOTOSense) is measuring part of the surface from the front of the torch position at a measuring distance of 150 mm. Connecting an iron pipe of 200 mm in diameter with a hollow-casted adapter in a V groove seam configuration requires adaptive welding in terms of robot positioning and changing of welding parameters in multilayer welding. The thickness of the walls is 10 mm, which results in five layers, including an adjustable amount of deposited material regarding the gap size between both parts. By adapting the robot speed based on the measured gap (Figure 7B), the system can meet the constant fill requirement.

The part is scanned along the entire circumference before welding to locate all the layers and define the speed of welding, provided by the synchronized motion between the robot and external axis. The root layer is welded with the same parameters while using a bead weld support ring to hold on to the melted material and create constant conditions (Figure 7C). To eliminate overlapping of all welds in the same location, each weld start/end position is shifted by one section (45°) in the direction of welding. The achieved tolerances of the welded geometry are <0.5 mm and can be inspected by the same measuring system at the end of the welding process.

### Laser Welding

**FIGURE 8:**
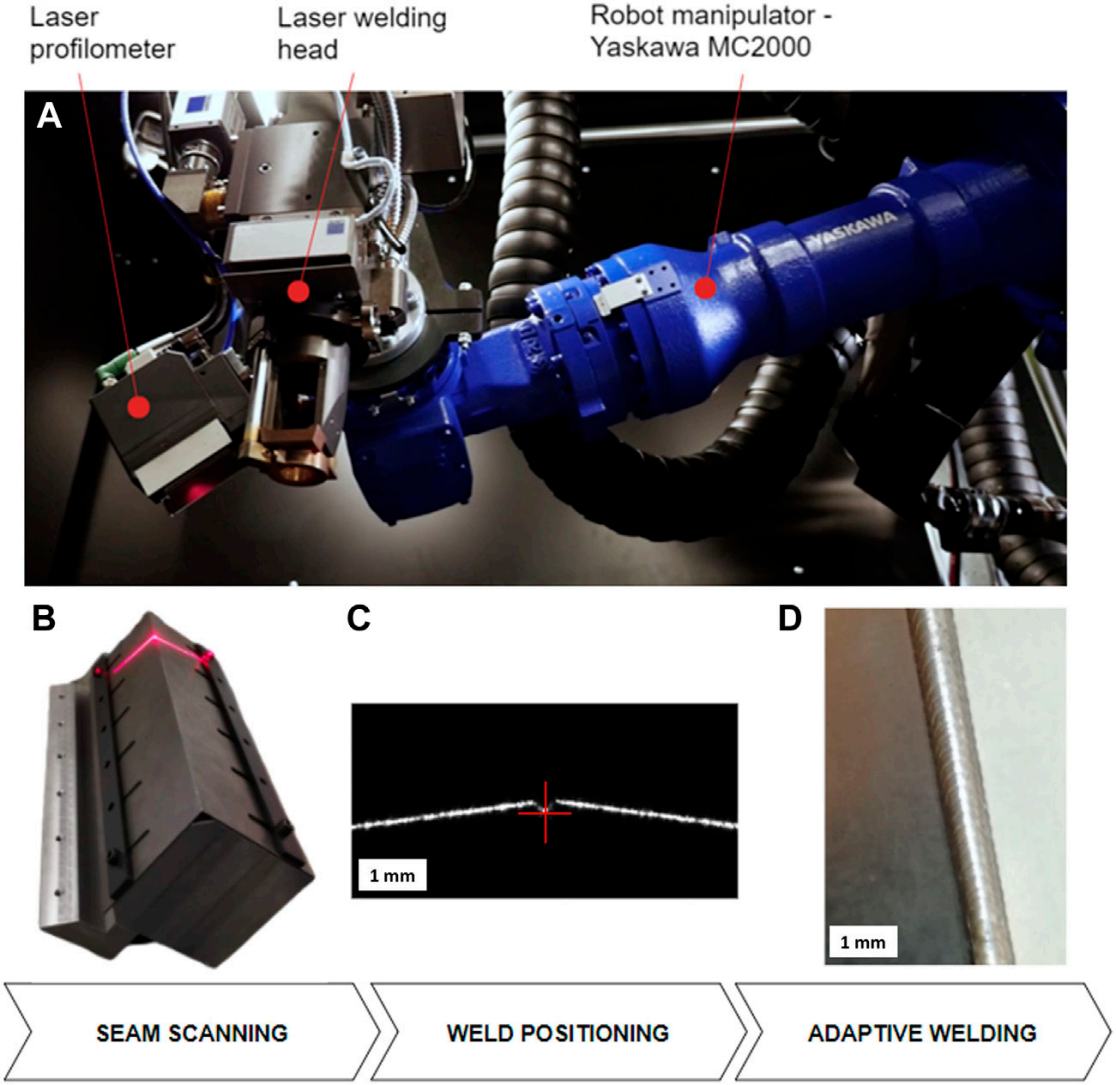
Example of the adaptive laser system **(A)** for welding of thin steel plates. The process is composed of seam 3D scanning **(B)**, detection of the welding edge **(C)**, and laser welding along the detected path **(D)**.

Figure 8 shows a laser welding system equipped with a 4-kW fiber laser (Trumpf, TruDisk 4,000), a fixed focusing laser head (Trumpf, BEO D35), and a laser profilometer (Yaskawa MOTOSense) attached to a robot arm (Yaskawa MC2000 high precision). The working distance of the laser head is 200 mm. The pose of the laser profilometer is chosen so that the center of the measuring range coincides with the focus of the welding laser. In this way, the focus position can be identified when the laser line from the profilometer is aligned with the pilot beam of the welding head. This simplifies the visual inspection of the robot’s positioning since there is no physical teaching point.

The system is designed to handle small series production of thin steel and aluminum sheet parts (thickness up to 2 mm) in simplified jigs to reduce the preparation costs for each batch of parts. The positioning precision of the system is better than 0.06 mm, which satisfies the tolerance condition for laser welding with the focus diameters down to 0.12 mm. Automatic teaching of welding trajectory is approximately four times more precise than visual teaching.

## Conclusion

An automatic calibration of an adaptive 3D scanner-based robot welding system was developed. It is based on the scanning of the reference geometry from multiple poses, the detection of reference points, and the numerical minimization of the deviation between the measured and the reference points. The method enables robust and precise calibration of all transformation parameters (hand-eye and intrinsic). The accuracy of the robotic welding system after the calibration is better than 0.06 mm perpendicular to the scanning direction. This enables the use of such systems not only in conventional arc welding applications but also in high-precision laser welding. Besides precise teaching of welding trajectories, such systems can also be used for adaptive multilayer welding and simultaneous post-process inspection of welded geometry.

An important feature of this calibration procedure is also completely automated and has fast processing times (approximately 90 s). This enables its implementation into a regular daily plan of robot self-maintenance and monitoring.

## Data Availability

The raw data supporting the conclusion of this article will be made available by the authors, without undue reservation.
